# A Case of Pathological Complete Response after “Conversion Surgery” for Initially Unresectable Locally Advanced Intrahepatic Cholangiocarcinoma

**DOI:** 10.70352/scrj.cr.25-0569

**Published:** 2026-01-08

**Authors:** Yasunori Shirakawa, Tomoaki Yoh, Takashi Ito, Satoshi Ogiso, Takamichi Ishii, Masakazu Fujimoto, Hironori Haga, Etsuro Hatano

**Affiliations:** 1 Department of Surgery, Graduate School of Medicine, Kyoto University, Kyoto, Kyoto, Japan; 2 Department of Diagnostic Pathology, Graduate School of Medicine, Kyoto University, Kyoto, Kyoto, Japan

**Keywords:** intrahepatic cholangiocarcinoma, pathological complete response, conversion, pembrolizumab, gemcitabine, cisplatin plus S-1 (GCS)

## Abstract

**INTRODUCTION:**

Intrahepatic cholangiocarcinoma (iCCA) is the second most common liver cancer and has a poor prognosis. Given the recent advancements in drug therapy, the topic of so-called “conversion surgery” in biliary tract cancer, including iCCA, is evolving; however, only a few cases have been reported.

**CASE PRESENTATION:**

A 50-year-old female was referred to our hospital for a liver tumor identified on abdominal ultrasonography. She was diagnosed with iCCA based on tumor biopsy. Due to extensive vascular and bile duct invasion, iCCA was initially considered unresectable. After 8 cycles of gemcitabine, cisplatin plus S-1 (GCS) therapy, CT revealed a partial response. Considering that microsatellite instability–high (MSI-H) was detected, we switched the regimen from GCS to pembrolizumab. However, after 1 cycle of pembrolizumab therapy, immune checkpoint inhibitor (ICI)–induced hepatitis was suspected; therefore, pembrolizumab therapy was suspended. GCS therapy was restarted, and after another 3 cycles, the iCCA was deemed resectable; therefore, conversion surgery was performed. Postoperative histopathological examination revealed a pathological complete response (pCR), and the patient remained alive more than 5 years postoperatively without recurrence or metastasis.

**CONCLUSIONS:**

We experienced a case of pCR induced by GCS chemotherapy and pembrolizumab monotherapy. Although the direct contribution of pembrolizumab remains unclear, a possible synergistic effect with GCS chemotherapy was suggested, particularly in MSI-H tumors.

## Abbreviations

ALT
: alanine aminotransferase
AST
: aspartate aminotransferase
B4
: bile duct 4
BTC
: biliary tract cancer
CA19-9
: carbohydrate antigen 19-9
CEA
: carcinoembryonic antigen
CECT
: contrast-enhanced CT
ERBD
: endoscopic retrograde biliary drainage
ERCP
: endoscopic retrograde cholangiopancreatography
EUS-FNA
: endoscopic ultrasound-guided fine-needle aspiration
FDG
: fluorodeoxyglucose
GC
: gemcitabine plus cisplatin
GCD
: gemcitabine, cisplatin plus durvalumab
GCP
: gemcitabine, cisplatin plus pembrolizumab
GCS
: gemcitabine, cisplatin plus S-1
HCC
: hepatocellular carcinoma
iCCA
: intrahepatic cholangiocarcinoma
ICG-Krem
: indocyanine green clearance of remnant liver
ICI
: immune checkpoint inhibitor
irAE
: immune-related adverse event
MDT
: multidisciplinary team
MSI-H
: microsatellite instability–high
pCR
: pathological complete response
PCR
: polymerase chain reaction
PTPE
: percutaneous transhepatic portal vein embolization

## INTRODUCTION

iCCA is the second most common primary liver cancer after HCC, and has shown an increasing global mortality trend.^1)^ Surgical resection remains one of the most effective treatments for iCCA^2)^; however, its applicability is often limited due to the advanced stage of diagnosis. Even when surgical resection is possible, the high recurrence rate highlights the necessity of a multidisciplinary treatment approach.^3)^

Given these limitations, chemotherapy has become an important component of multidisciplinary strategies, offering the possibility of tumor downstaging and conversion to resectability. In recent years, several new drug regimens have been widely adopted for unresectable BTC, including iCCA, demonstrating significant clinical efficacy.^4–6)^ With advances in drug therapy, an increasing number of cases of BTC that were initially unresectable but converted to resectable following chemotherapy have been reported,^7,8)^ and the significance of conversion surgery has been increasingly recognized.^9–11)^ However, the literature on such cases remains limited.

Herein, we present the case of a patient who underwent GCS chemotherapy combined with pembrolizumab, followed by surgical resection. Moreover, this case showed a pCR.

## CASE PRESENTATION

**Fig. 1:**
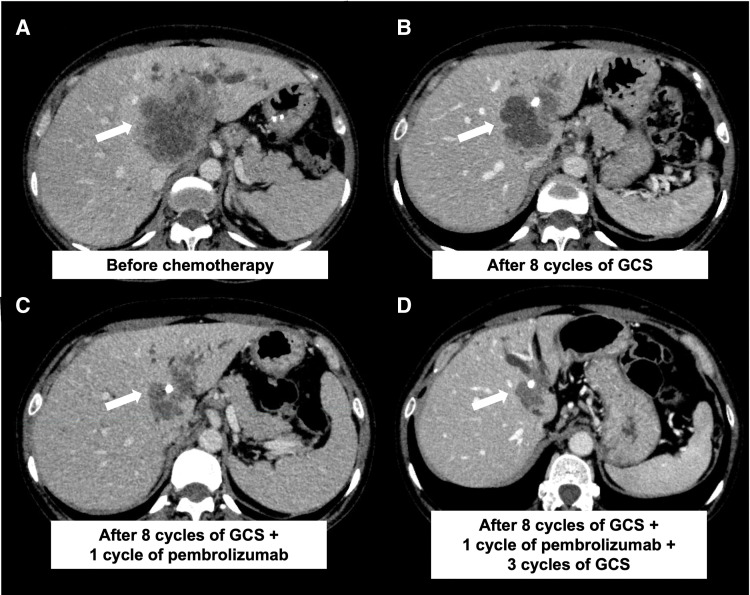
Abdominal CECT during chemotherapy. (**A**) Before starting chemotherapy, CECT showed a 6.5 × 5.2 cm tumor (arrow). (**B**) After 8 cycles of GCS chemotherapy, CECT showed a 5.0 × 3.4 cm tumor (arrow). (**C**) After 1 cycle of pembrolizumab therapy, CECT showed a 4.9 × 3.0 cm tumor (arrow). (**D**) After another 3 cycles of GCS therapy, CECT showed a 2.7 × 2.1 cm tumor (arrow). CECT, contrast-enhanced CT; GCS, gemcitabine, cisplatin plus S-1

**Fig. 2:**
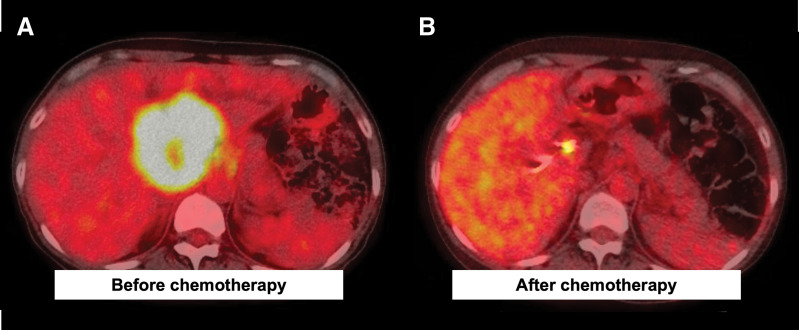
FDG-PET/CT during chemotherapy. (**A**) Before starting chemotherapy, FDG-PET/CT showed intense uptake (SUVmax = 12.6) in the liver. (**B**) After 11 cycles of GCS chemotherapy and 1 cycle of pembrolizumab therapy, FDG-PET/CT showed a remarkable reduction in both the intensity and extent of FDG uptake. The residual FDG uptake was presumed to represent reactive or inflammatory changes rather than residual tumor activity. FDG, fluorodeoxyglucose; GCS, gemcitabine, cisplatin plus S-1

**Fig. 3:**
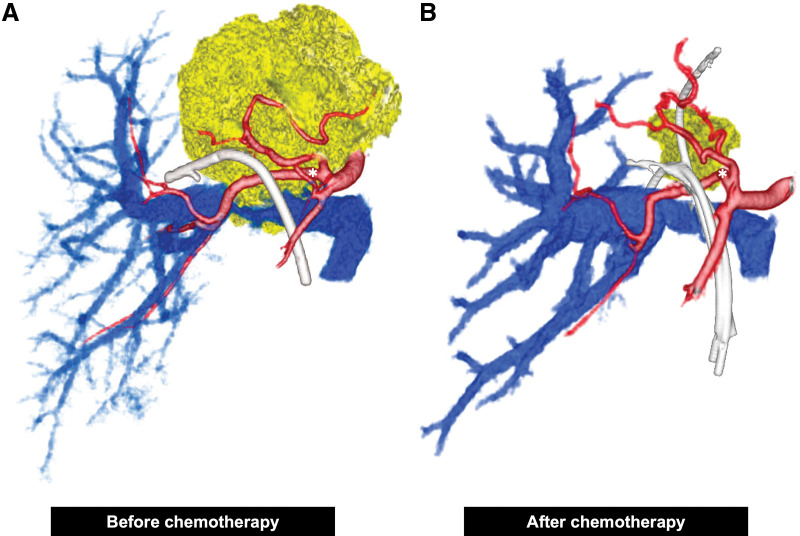
3D reconstruction images during chemotherapy using SYNAPSE VINCENT (FUJIFILM, Tokyo, Japan). The hepatic artery, portal vein, tumor, and ERBD tubes are shown in red, blue, yellow, and white, respectively. (**A**) Before starting chemotherapy, the proximal right hepatic artery (asterisk) was in contact with the tumor, suggesting tumor invasion. (**B**) After chemotherapy, the tumor shrank remarkably, with minimal contact with the proximal right hepatic artery (asterisk). ERBD, endoscopic retrograde biliary drainage

A 50-year-old female patient presented to a referring hospital with epigastric pain, and abdominal ultrasonography revealed a liver tumor. Because jaundice was also observed, ERBD tubes were placed in the right anterior hepatic duct and B4. She was subsequently referred to our hospital. Initial laboratory data showed liver dysfunction, with AST and ALT levels of 109 U/L and 103 U/L, respectively. Serum tumor markers were normal as follows: CEA 2.5 ng/ml (normal range 0–5 ng/mL) and CA19-9 17.9 U/mL (normal range 0–37 U/mL). CECT revealed a 6.5 × 5.2 cm low-density liver mass mainly occupying segment IV (**Fig. 1**) and enlarged paraaortic lymph nodes that showed no malignancy on EUS-FNA. FDG-PET/CT revealed intense uptake (SUVmax = 12.6) in the liver, corresponding to the low-density mass observed on CECT (**Fig. 2**). The confluence of the right and left hepatic ducts was obstructed, and the confluence of the right anterior and posterior hepatic ducts was invaded. The proximal right hepatic artery was in contact with the tumor, suggesting invasion, whereas the posterior branch was intact (**Fig. 3**). Forceps biopsy specimens from the hilar bile duct revealed poorly differentiated adenocarcinoma. Therefore, the patient was diagnosed with iCCA (cT3N0M0, cStage III).

**Fig. 4:**
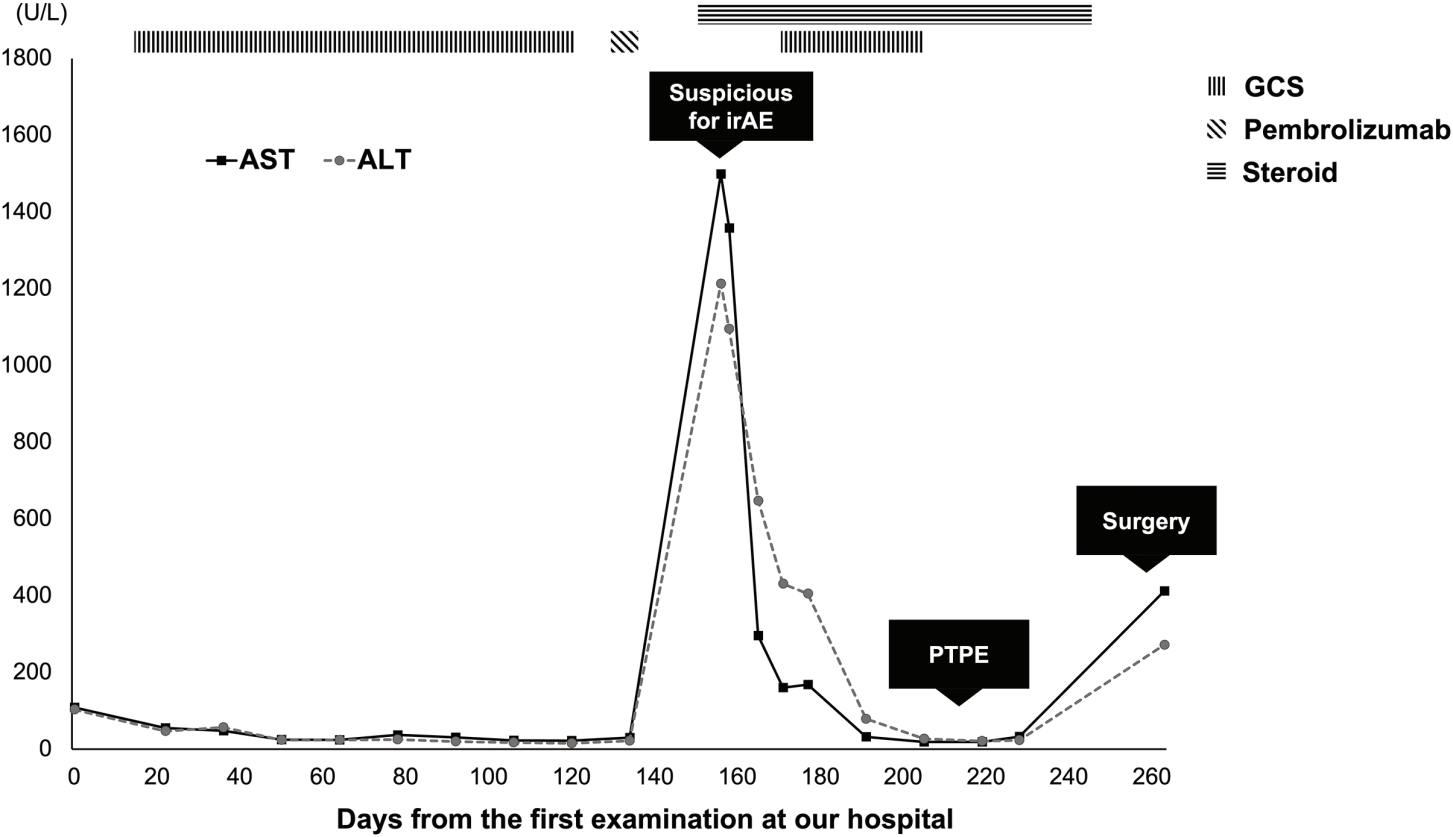
AST and ALT levels during chemotherapy. AST and ALT levels remained stable during 8 cycles of GCS chemotherapy. However, these levels increased dramatically after 1 cycle of pembrolizumab therapy. Immune-mediated hepatitis, an irAE, was suspected; therefore, pembrolizumab therapy was suspended, and steroid pulse therapy (methylprednisolone 50 mg/day) was initiated. Subsequently, the levels decreased, and GCS chemotherapy was resumed. After 3 cycles of GCS chemotherapy, preoperative PTPE was performed. AST and ALT levels remained stable preoperatively but slightly increased postoperatively. ALT, alanine aminotransferase; AST, aspartate aminotransferase; GCS, gemcitabine, cisplatin plus S-1; irAE, immune-related adverse event; PTPE, percutaneous transhepatic portal vein embolization

Radical surgical resection combined with vascular and bile duct resections was planned. However, reconstruction was considered difficult because of extensive vascular and bile duct invasion. Consequently, the iCCA was regarded as an unresectable locally advanced tumor, and GCS chemotherapy was planned. Gemcitabine and cisplatin were administered intravenously at doses of 1000 and 25 mg/m^2^, respectively, on day 1, and S-1 was administered orally at a dose of 50 mg twice a day for 7 consecutive days, repeated every 2 weeks. After 8 cycles of GCS therapy, CECT showed a partial response to chemotherapy. PCR of the liver tumor biopsy specimen showed MSI-H; therefore, we switched the regimen from GCS to pembrolizumab. Pembrolizumab was planned to be administered intravenously at a dose of 200 mg every 3 weeks. After 1 cycle of pembrolizumab therapy, CECT showed a partial response to chemotherapy: further shrinkage of the liver mass. However, the patient complained of right upper quadrant pain, and laboratory data showed elevated liver enzymes as follows: AST 1499 U/L and ALT 1213 U/L (**Fig. 4**). After ruling out cholangitis using ERCP, ICI-induced hepatitis was suspected; therefore, pembrolizumab therapy was suspended, and steroid pulse therapy (methylprednisolone 50 mg/day) was initiated. One week later, the dose of methylprednisolone was reduced to 40 mg/day, and after another 1 week, laboratory data showed lower liver enzymes as follows: AST 160 U/L and ALT 431 U/L. Steroid pulse therapy was considered effective, and GCS therapy was restarted. After another 3 cycles of GCS therapy, CECT showed a partial response to chemotherapy with further shrinkage of the liver mass, and FDG-PET/CT showed a remarkable reduction in both the intensity and extent of FDG uptake. The proximal right hepatic artery exhibited minimal contact with the tumor; therefore, arterial reconstruction was considered to be safe. Surgical resection was planned approximately 8 months after the initiation of the first cycle of chemotherapy. As the left portal vein was already occluded, preoperative PTPE of the right anterior branch of the portal vein was performed to increase the volume of the posterior segment. As a result, it increased from 32.9% of the entire liver to 37.6%, and the ICG-Krem increased from 0.0697 to 0.0722.

**Fig. 5:**
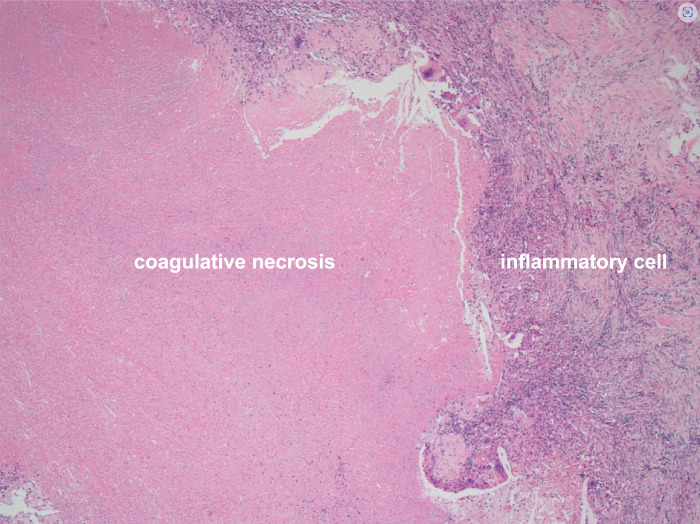
Pathological findings of the surgically resected specimen. The tumor exhibited extensive coagulative necrosis surrounded by a fibrotic stroma with prominent mononuclear inflammatory cell infiltration, including scattered foreign body-type giant cells.

The iCCA was deemed resectable, and left hepatic trisegmentectomy concomitant with right portal vein and artery resection, regional lymphadenectomy, and hepaticojejunostomy were performed. Postoperative histopathological examination of the surgically resected specimen revealed pCR (**Fig. 5**). On POD 7, CT showed free fluid in the dissected section of the liver; therefore, biliary leakage was suspected, and percutaneous abscess drainage was performed (ISGLS grade B and Clavien–Dindo Grade IIIa). No other complications were observed, and the patient was discharged on POD 69. The MDT meeting determined that adjuvant chemotherapy would not be performed in this case because pCR had already been achieved. She remains alive more than 5 years postoperatively without recurrence or metastasis.

## DISCUSSION

In the era of advances in drug therapy for unresectable iCCA, the significance of conversion surgery has been increasingly recognized.^9–13)^ Despite its importance, data on this topic remain limited. Meanwhile, major pathological response has been emphasized in other malignancies, such as HCC, in the setting of conversion therapy.^14,15)^ This is the first reported case of a patient with initially unresectable iCCA, who achieved a pCR following GCS chemotherapy and pembrolizumab monotherapy.

Although surgical resection is the only curative treatment for iCCA, most patients are diagnosed at an advanced stage and are not eligible for surgery.^2)^ In this case, the tumor was initially deemed unresectable because of extensive vascular and bile duct invasion. In such cases, chemotherapy is generally recommended.^16)^ Historically, GC was the standard first-line therapy.^17)^ Recent trials, including TOPAZ-1 and KEYNOTE-966, have demonstrated the survival benefits of adding durvalumab (GCD) or pembrolizumab (GCP) to GC therapy.^5,18)^ In Japan, the MITSUBA trial led to the recommendation of GCS as a first-line therapy.^4,19)^ Among these regimens, only GCS was approved at the time of treatment planning and was therefore selected. Pembrolizumab monotherapy was initiated after it was covered by national insurance for MSI-H tumors, in accordance with the patient’s wishes.

The definition of “conversion surgery” varies; the Japanese Liver Cancer Association describes it as surgery made anatomically possible by a good response to chemotherapy or achieving oncological stability. Although a clear definition is lacking in the published reports, the available data on BTC, including iCCA, suggest that conversion surgery is associated with benefits.^9–13)^ Among the factors contributing to conversion surgery, tumor downsizing is one of the most straightforward and critical concepts. In this context, the MITSUBA trial demonstrated the highest response rate (41.5%).^4)^ In the present case, initial GCS chemotherapy achieved significant tumor shrinkage with a partial response; however, conversion surgery was not feasible at that point. Although direct evidence is lacking, considering the high response rate of pembrolizumab in MSI-H tumors^20)^ and the known association between irAEs and better therapeutic outcomes^21,22)^ in other malignancies, it is plausible that pembrolizumab contributed to tumor shrinkage in this case, even after a single cycle. It was also possible that induction GCS therapy enhanced the immune response by promoting the release of tumor antigens.^23)^ It should be noted that this is merely a speculation, and pCR can also be achieved with GCS therapy alone. As GCS was continued after pembrolizumab, the contribution of GCS to the treatment effect cannot be ruled out.

The reported cases of pCR after chemotherapy for initially unresectable iCCA are summarized in **Table 1**.^7,8,24–33)^ The chemotherapy cycle prior to conversion surgery ranges from 4 to 12 months, highlighting the differences in tumor responses and patient conditions, which makes it difficult to determine the optimal timing of the conversion surgery. Therefore, as in our case, constant discussion and reassessment of surgical indications by the MDT are essential.^34)^ Some cases include chemotherapy combined with ICIs; however, they do not show long-term survival without recurrence. Only Robinson et al.^32)^ have reported a case of long-term survival after achieving pCR with GCP therapy in the United Kingdom. In Japan, our case is the first to demonstrate a long-term survival of more than 5 years without recurrence after achieving pCR following chemotherapy combined with an ICI. In both cases, irAEs occurred, leading to the discontinuation of pembrolizumab. Consequently, as previously mentioned, this may have resulted in favorable outcomes. In addition, in some malignancies, pCR following ICI therapy has been associated with excellent outcomes,^14,15,35)^ and a similar effect may have occurred in this patient. Further studies are warranted to explore this issue.

**Table 1:** Previous cases of initially unresectable iCCA that achieved pCR after “conversion surgery”

Case	Year	First author	Age, Sex	cStage	Chemotherapy	irAEs	Operation methods	Survival
1	2007	Slupski	33, M	IVB	Doxorubicin, cisplatin, 5-FU, IFN K (9 cycles)	–	Right hepatectomy	>30 months (No recurrence)
2	2015	Tran	67, M	IVB	GEM + oxaliplatin (4 cycles)	–	Left trisectionectomy, caudate lobectomy	>6 months (No recurrence)
					GEM + cisplatin (5 cycles)			
3	2015	Kato	NR	NR	GEM + cisplatin (NR)	–	Left trisectionectomy, caudate lobectomy	NR
4	2016	Matsubara	68, F	IVB	GEM + cisplatin + S-1 (12 cycles)	–	Extended right hepatectomy	>9 months (No recurrence)
5	2018	Tatsuguchi	72, M	IVB	GEM + S-1 (10 cycles)	–	Left hepatectomy	>7 years (No recurrence)
6	2021	Abudalou	47, M	IVB	GEM + cisplatin + nab-PTX (3 cycles)	NR	Extended left hepatectomy	>6 months
					Pembrolizumab (5 cycles)			
7	2022	Li	52, F	IVA	Nab-PTX + S-1 + PD-1 inhibitor (6 cycles)	No	Left trisectionectomy	>1 year (No recurrence)
8	2022	Sumiyoshi	66, F	III	GEM + S-1 (4 cycles)	–	Left trisectionectomy	>6 months (No recurrence)
9	2022	Zhang	36, M	IVA	GEM + cisplatin + S-1 + nab-PTX + camrelizumab (5 cycles)	No	NR	>1 month (No recurrence)
10	2024	Shimamaki	79, F	IVA	GEM + cisplatin (4 cycles)	–	Left hepatectomy, caudate lobectomy	>2 years (No recurrence)
11	2024	Robinson	49, M	IVB	Pembrolizumab + GEM + cisplatin (10 cycles)	Yes	Right anterior sectionectomy, right adrenalectomy	>38 months
					Pembrolizumab (2 cycles)			
12	2025	Fukuda	64, M	IVB	GEM + cisplatin (4 cycles)	No	Right hepatectomy	>10 months (No recurrence)
					Durvalumab + GEM + cisplatin (6 cycles)			
Present case	2026	Shirakawa	50, F	III	GEM + cisplatin + S-1 (11 cycles)	Yes	Left trisectionectomy	>56 months (No recurrence)
					Pembrolizumab (1 cycle)			

–, no use of immune checkpoint inhibitors; F, female; FU, fluorouracil; GEM, gemcitabine; iCCA, intrahepatic cholangiocarcinoma; IFN K, interferon K; irAEs, immune-related adverse events; M, male; nab-PTX, nanoparticle albumin-bound paclitaxel; NR, not reported; pCR, pathological complete response

## CONCLUSIONS

We experienced a case of pCR induced by GCS chemotherapy and pembrolizumab monotherapy, confirmed by surgical resection. Although the direct contribution of pembrolizumab remains unclear, the long-term recurrence-free survival and evidence from other malignancies suggest a possible synergistic effect with GCS chemotherapy, particularly in MSI-H tumors. Accumulation of more cases and further discussion are needed to clarify the role of ICIs in conversion strategies for advanced iCCA.

## DECLARATIONS

### Availability of data and materials

The dataset supporting the conclusions of this article is included within the article.
